# Dietary purslane modulates gut microbiota and fecal metabolites in aging rats

**DOI:** 10.3389/fmicb.2025.1549853

**Published:** 2025-03-19

**Authors:** Jingwen Deng, Xia Wang, Can Yan, Zicheng Huang, Hui Luo, Caihua Dai, Xiaoliu Huang, Yushan Huang, Qiang Fu

**Affiliations:** ^1^Affiliated Hospital of Jinggangshan University, Ji’an, China; ^2^Jiangxi Province Key Laboratory of Organ Development and Epigenetics, Clinical Medical Research Center, Affiliated Hospital of Jinggangshan University, Medical Department of Jinggangshan University, Ji'an, China; ^3^Department of Pathology, Ji'an Central People's Hospital, Ji'an, China; ^4^College of Mathematics and Physics, Xinjiang Agricultural University, Urumqi, China; ^5^Center for Evidence Based Medical and Clinical Research, First Affiliated Hospital of Gannan Medical University, Ganzhou, China

**Keywords:** gut microbiota, metabolites, intestinal health, aging rats, purslane

## Abstract

**Introduction:**

*Portulaca oleracea L.* (purslane) is a highly nutritious and edible wild vegetable beneficial to human health. However, its impacts on the structure of gut microbiota and fecal metabolites in aging individuals remain unclear. This study aims to clarify its potential mechanisms in aging-related gut health.

**Methods:**

Naturally aged rats (18 months) were divided into two groups. One group was fed a maintenance chow, and the other was fed a mixture with 3.5% purslane for 15 weeks. Hematoxylin-eosin staining, gas chromatography-mass spectrometry, and 16S rDNA high-throughput sequencing were employed to explore the effects of purslane on the intestinal health of these rats.

**Results:**

The fecal concentrations of acetic acid, propionic acid, butyric acid, valeric acid, caproic acid, and total short-chain fatty acids (SCFAs) were significantly increased in aging rats fed the purslane supplement. Purslane significantly reduced the relative abundance levels of Firmicutes and Fusobacteria, as well as the ratio of Firmicutes to Bacteroidetes. KEGG pathway analysis annotated 109 differential metabolites, which mainly affected metabolic pathways such as linoleic acid metabolism, arachidonic acid metabolism, primary bile acid biosynthesis, steroid biosynthesis, and steroid hormone biosynthesis. There was a strong correlation between *Paracbacteroides*, the *Prevotella* NK3B31_group, the *Rikenella*_RC9_gut_group, and SCFA levels. Aging rats consuming purslane had a more complete and healthy gut morphology than the control group.

**Discussion:**

These results suggested that the maintenance of intestinal health by purslane in aging rats might be associated with the targeted regulation of gut microbiota and fecal metabolites.

## 1 Introduction

Purslane, belonging to the *Portulaca* family, is an annual succulent herb that is widely distributed throughout the world (Zhou et al., 2015). It is known for its multiple health benefits, including antibacterial, anti-inflammatory, antioxidant, and wound-healing properties, and has been dubbed as a “global panacea” and the “vegetable for long life” by many health writers (Zhou et al., 2015). Purslane is rich in polysaccharides, flavonoids, terpenoids, organic acids, coumarins, and alkaloids. These compounds have been extensively studied and have been reported to have various beneficial effects, including antitumor, hypoglycemic, immunity-enhancing, and cardiovascular protection properties (He et al., 2021; Jalali and Ghasemzadeh Rahbardar, 2022). Studies have shown that purslane extract can effectively relieve the clinical symptoms of atopic dermatitis and is a potentially effective drug for the treatment of atopic dermatitis-like diseases (Lv et al., 2022). In addition, purslane has been reported to regulate intestinal flora and metabolites, thereby benefiting health (Li et al., 2024). For instance, recent studies have also revealed that purslane can promote the growth of broilers by regulating their gut microbiota (Wang et al., 2021). Purslane has been proven to effectively inhibit coccidia growth, increase the relative abundance of beneficial bacteria, and maintain the homeostasis of intestinal flora in the intestinal tracts of mammals, such as Hu lambs, thus improving the digestibility of nutrients (Li et al., 2024).

It has been reported that 20% of the world’s population will be over 65 years old by 2050, and the number of elderly people aged over 65 and 80 years in China will reach 400 million and 150 million, respectively (Shilpa Dogra et al., 2022; Fang et al., 2015). With increasing age, the risks of associated diseases also rise, including cancer, type 2 diabetes, cardiovascular disease, and neurological diseases, which pose significant healthcare challenges (Bruins et al., 2019; Pyo et al., 2020). Research has shown that normal aging is associated with a series of complex physiological processes and the loss of anatomical structure (da Costa et al., 2016). The gut microbiome plays an important role in healthy aging and ultimately affects host longevity (Ling et al., 2022). For example, gut microbiota regulates various host metabolic processes, such as lipid metabolism, glucose metabolism, and energy homeostasis (Schoeler and Caesar, 2019). Further, gut microbial dysbiosis and microbial metabolite changes are associated with a variety of age-related diseases, including Parkinson’s disease (Sun and Shen, 2018), Alzheimer’s disease, and multiple sclerosis (Mou et al., 2022). Short-chain fatty acids (SCFAs), which are produced by intestinal flora via the fermentation of dietary fiber or indigestible carbohydrates, are important energy and signaling molecules (Morrison and Preston, 2016; Koh et al., 2016). Lee et al. found that SCFAs derived from intestinal microbes could promote recovery after a stroke in aged mice (Lee et al., 2020). Diet has been widely recognized as a major factor affecting the composition and function of human gut microbiota. A comparative study showed that African children on a fiber-rich diet had more Bacteroidetes and SCFAs and fewer Firmicutes compared to a group of European children (*p* < 0.001), while European children on a typical Western diet had significantly increased levels of Enterobacteriaceae (*Shigella* and *Escherichia coli*) (Coman and Vodnar, 2020). However, there is currently a gap in the research regarding the impact of purslane on the gut microbiota and metabolites of aging individuals, and the effects of fiber-rich purslane on the gut microbiota and related metabolites in the elderly remain unclear.

This study utilized naturally aged rats as a model to thoroughly investigated the regulatory effects of purslane on the structure of gut microbiota and fecal metabolites, aiming to fill this research void and offering a theoretical foundation and dietary intervention basis for promoting healthy aging.

## 2 Materials and methods

### 2.1 Materials and reagents

Purslane powder was purchased from Lanzhou Wotelaisi Biotechnology Co., Ltd. (Wotls, Lanzhou, China). Pellet feed for rats was purchased from Hunan SJA Laboratory Animal Co., Ltd. [Production license no.: SCXK(Xiang)2014–0002]. Purslane-added feed was prepared by mixing 96.5% experimental rat maintenance pellet feed with 3.5% purslane dry powder (Hunan SJA Laboratory Animal Co., Ltd.). A DNeasy Power Soil Kit and a QIAamp 96 Power Fecal QIAcube HT Kit were purchased from QIAGEN (Germany). A Qubit dsDNA Assay Kit and Tks Gflex DNA Polymerase were purchased from Life Technologies (USA) and Takara (Japan), respectively. Acetic acid (Ace), propionic acid (Pro), butyric acid (But), pentanoic acid (Pen), hexanoic acid (Hex), isobutyric acid (i-But) and isovaleric acid (i-Pen) standards (HPLC grade) were purchased from Sigma Corporation. All other chemicals and solvents used were analytically pure or of chromatographic grade.

### 2.2 Animals and diet

**Figure 1:**
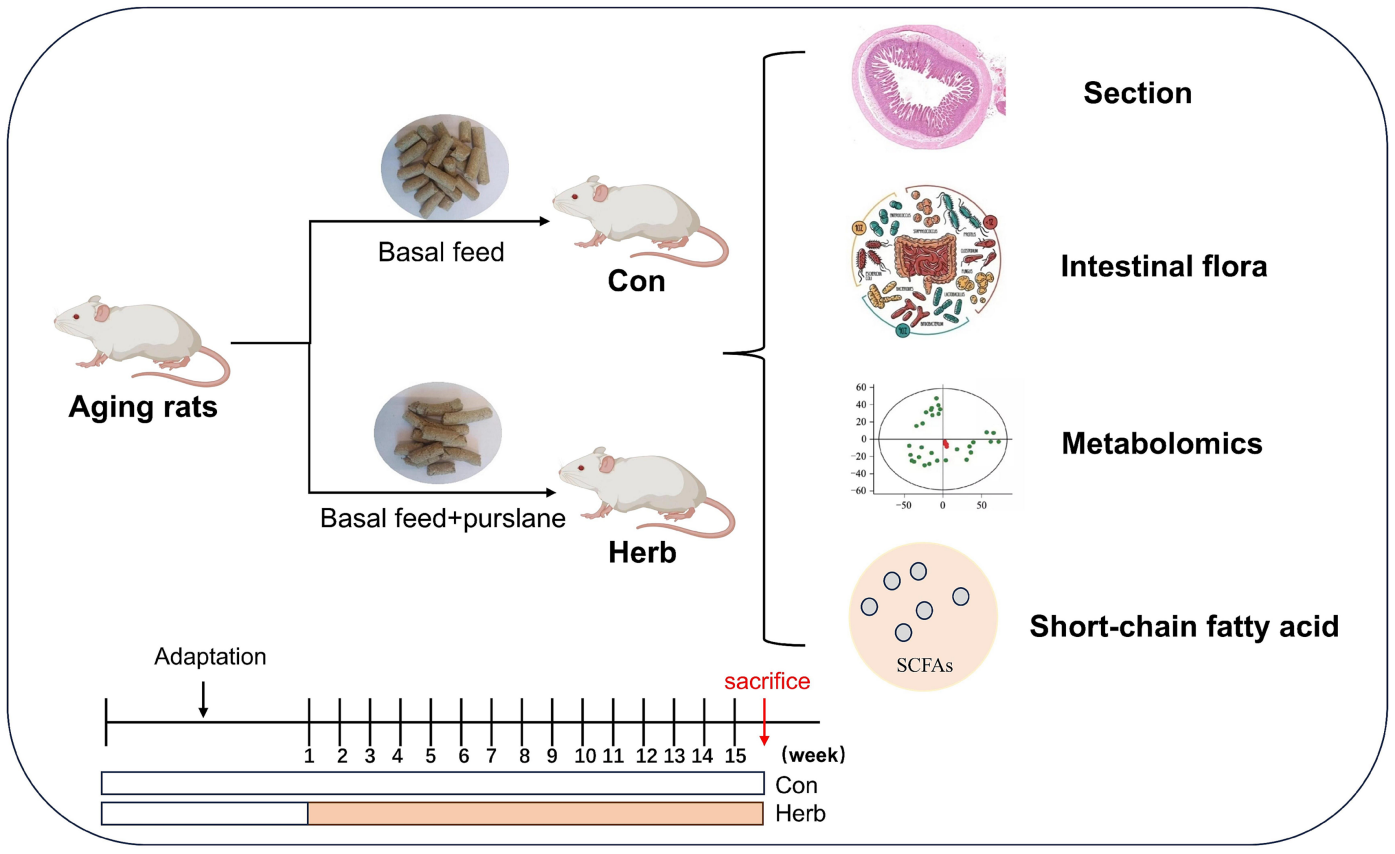
Flow chart of experimental design. Animals were sacrificed by cervical dislocation under deep anesthesia with isoflurane.

Twenty 18-month-old specific pathogen-free (SPF) Sprague–Dawley rats were purchased from Hunan SJA Laboratory Animal Co., Ltd. [laboratory animal production license: SCXK(Xiang)2019–0004]. The rats were allowed free access to food in the Experimental Animal Center of Jinggangshan University under SPF conditions (SYXK<Jiangxi>2023–0009) at 23 ± 1°C, 50–60% relative humidity, with alternating 12-h light/dark cycles. After one week of adaptive feeding, all animals (*n* = 10 per group) were randomly divided into a control group (fed laboratory mice maintenance pellets; Con) and a purslane group (fed laboratory mice maintenance pellets containing 3.5% purslane; Herb) for 15 weeks. All animal use procedures were approved by the Animal Care and Use Committee of Jinggangshan University. The flow chart of experimental design was shown in Figure 1.

### 2.3 Methods

#### 2.3.1 Sample collection and preservation

The day before the end of the animal experiment, the animals were handled under sterile conditions, and their anuses were squeezed to stimulate feces. The feces were then collected in sterile frozen tubes, quick-frozen in liquid nitrogen and stored at −80°C. These samples were used for analyzing SCFAs, intestinal flora and fecal metabolites. At the end of the animal experiment, the animals were anesthetized and killed; ileum and colon tissue samples were taken and fixed in paraformaldehyde for histomorphological analysis.

#### 2.3.2 Histological analysis

The tissues were fixed in 4% paraformaldehyde for over 48 h, and paraffin embedding was performed. The tissues were cut into sections, and the sections were successively dewaxed, hydrated, hematoxylin–eosin (H&E) stained, dehydrated and sealed. The tissue sections were observed under an optical microscope, and images were recorded.

#### 2.3.3 Analysis of SCFA yield

First, 150 mg of feces was dissolved in 1 mL of 5 mmol/L NaOH solution. After homogenization and ultrasonic extraction at low temperature, the supernatant was collected following centrifugation and then derivatized at 12,000 rpm and 4°C. The derivatized metabolites were extracted and analyzed using gas chromatography–mass spectrometry (GC–MS) (7890B-5977A GC/MSD, Agilent Technologies Inc., CA, USA). Chromatography was performed on a DB-5MS capillary column (30 m × 0.25 mm × 0.25 μm; Agilent J&W Scientific, Folsom, CA, USA) at an initial temperature of 50°C for 5.2 min. The temperature was then increased with 10°C/min to 70°C for 1.3 min; raised with 3°C/min to 85°C for 1 min; raised with 5°C/min to 110°C for 1 min; and raised with 30°C/min to 290°C for 9 min. Helium was used as the carrier gas at a flow rate of 1.0 mL/min. The mass spectrum conditions were as follows: electron bombardment ion source (EI), ion source temperature 230°C, quaternary bar temperature 150°C and electron energy 70 eV. The full scanning mode (SCAN) was used, and the quality scanning range was 30–600 m/z. The results were analyzed using MassHunter software (Agilent Inc., version B.07.01) and an Agilent chemical workstation.

#### 2.3.4 Analysis of intestinal flora

Feces were collected from the colon and sent to OE Biotech Co., Ltd. (Shanghai, China) for 16S rRNA gene sequencing (100 mg). Specifically, total genomic DNA was extracted from each sample according to the instructions given on the MagPure Soil DNA LQ Kit (Magan). The V3–V4 hypervariable regions (343F 5’-TACGGRAGGCAGCAG-3′ and 798R 5′ -AGGGTATCTAATCCT-3′) of prokaryotic 16S rRNA were selected for amplification and bacterial diversity analysis. The raw data were read and primers were cut out using Cutadapt software. Using the DADA2 algorithm, qualified double-ended raw data were analyzed according to the default parameters of QIIME 2 (V2020.11) for quality control, such as quality filtering, noise reduction, splicing and dechimerism. The final, valid data were used for further bioinformatics analysis.

#### 2.3.5 Liquid chromatography mass spectrometry (LC–MS)/MS detection

For this step, 30 mg of each sample was added to 400 μL of methanol–water (*V:V* = 4:1), and was precooled at −40°C for 2 min. The samples were then ground (60 Hz, 2 min) and extracted via ultrasound in an ice water bath for 10 min, left at −40°C overnight and then centrifuged for 10 min (12,000 rpm, 4°C). After drying, 300 μL of the supernatant was added to 300 μL of methanol–water (*V:V* = 4:1) for redissolution. This was vortexed for 30 s, placed in an ultrasonic ice water bath for 3 min, left to stand at −40°C for 2 h and then centrifuged for 10 min (12,000 rpm, 4°C). The supernatant was filtered using a 0.22 μm filter and analyzed using an ultra-high performance liquid series high-resolution mass spectrometer (Dionex U3000 UHPLC/QE Plus, Thermo Fisher Technologies). Separation was performed on an ACQUITY UPLC HSS T3 (100 mm × 2.1 mm, 1.8 μm) column (Waters) at 45°C. The mobile phase consisted of eluent A (containing 0.1% formic acid water) and eluent B (containing 0.1% formic acid acetonitrile) at a flow rate of 0.35 mL/min.

#### 2.3.6 Metabolite analysis

Baseline filtration, peak recognition, integration, retention time correction, peak alignment and normalization to total spectral intensity of the original data were performed using Progenesis QI v2.3 software (Nonlinear Dynamics, Newcastle, UK). Compound identification was based on precise mass, secondary fragments, and isotopic distribution. The metabolites were annotated based on Kyoto Encyclopedia of Genes and Genomes (KEGG) and characterized using the Human Metabolome Database (HMDB), LIPID MAPS (v2.3), METLIN database and a self-built database. Then, based on KEGG^1^, HMDB^2^ and LIPID MAPS^3^, the metabolites were annotated. VIP > 1 and *p* < 0.05 were used to identify the differentially expressed metabolites of the two groups, respectively.

#### 2.3.7 Statistical data analysis

The experimental data were all presented as mean ± SD values. Comparisons between two groups were conducted using the *t*-test for two independent samples. Pearson’s correlation analysis was conducted between SCFAs and different bacteria as well as different metabolites. *p* < 0.05 indicated statistically significant differences. Statistical plots were drawn using GraphPad Prism 8.2 (GraphPad Software Inc., San Diego, CA, USA).

## 3 Results

### 3.1 Effects of purslane on the intestinal histological features of aging rats

**Figure 2:**
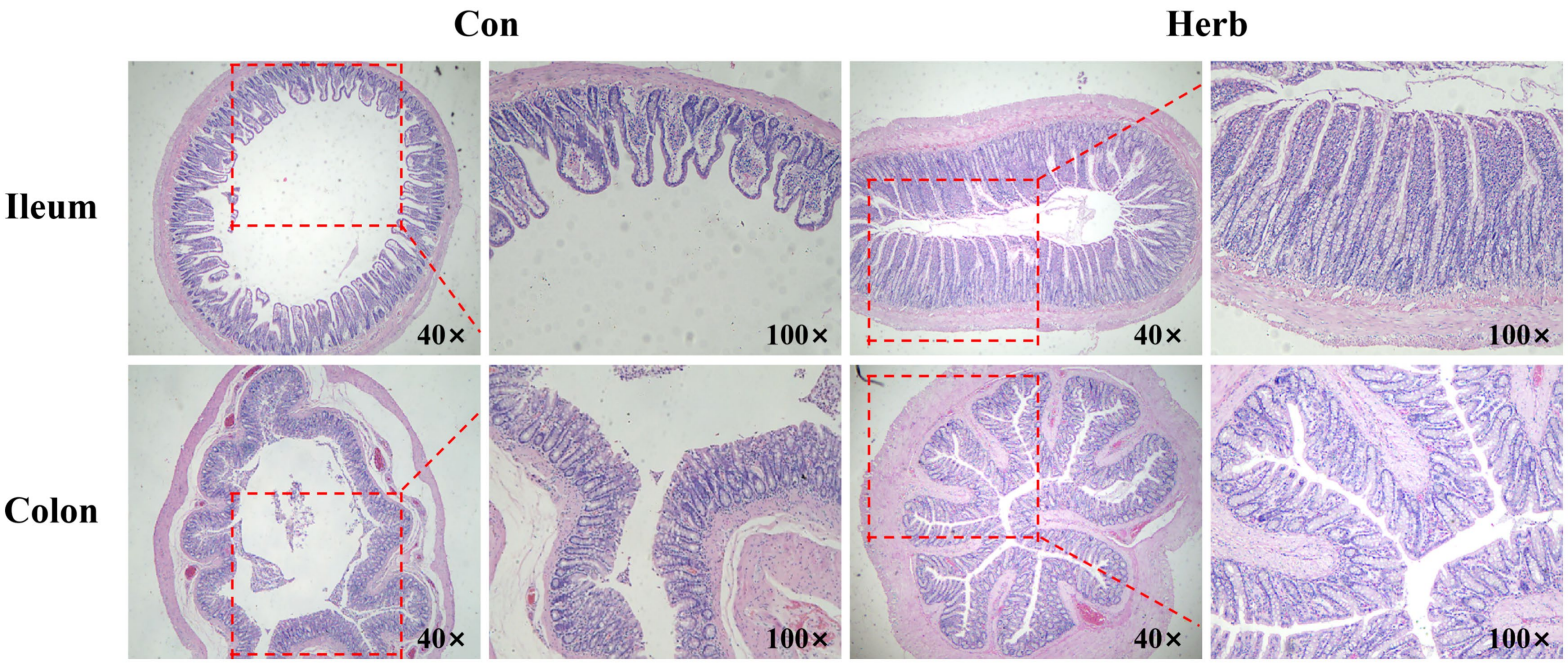
Representative images of the H&E staining of ileum and colon tissue.

The morphological characteristics of the intestinal tissue in aging rats clearly demonstrated age-related changes. The mucosal epithelial cells of the ileum and colon were shed, among which the ileum villi were unevenly distributed to a great degree and had decreased in number. In addition, the colon was irregular in shape, unclear in structure, and incomplete (Figure 2). In contrast, ileum and colon tissues from rats that received purslane supplementation maintained clear and structured morphology. The epithelial tissue and muscle layer remained intact, while the intestinal villi of the ileum were abundant and regularly arranged.

### 3.2 Effects of purslane on SCFAs in the feces of aging rats

**Figure 3:**
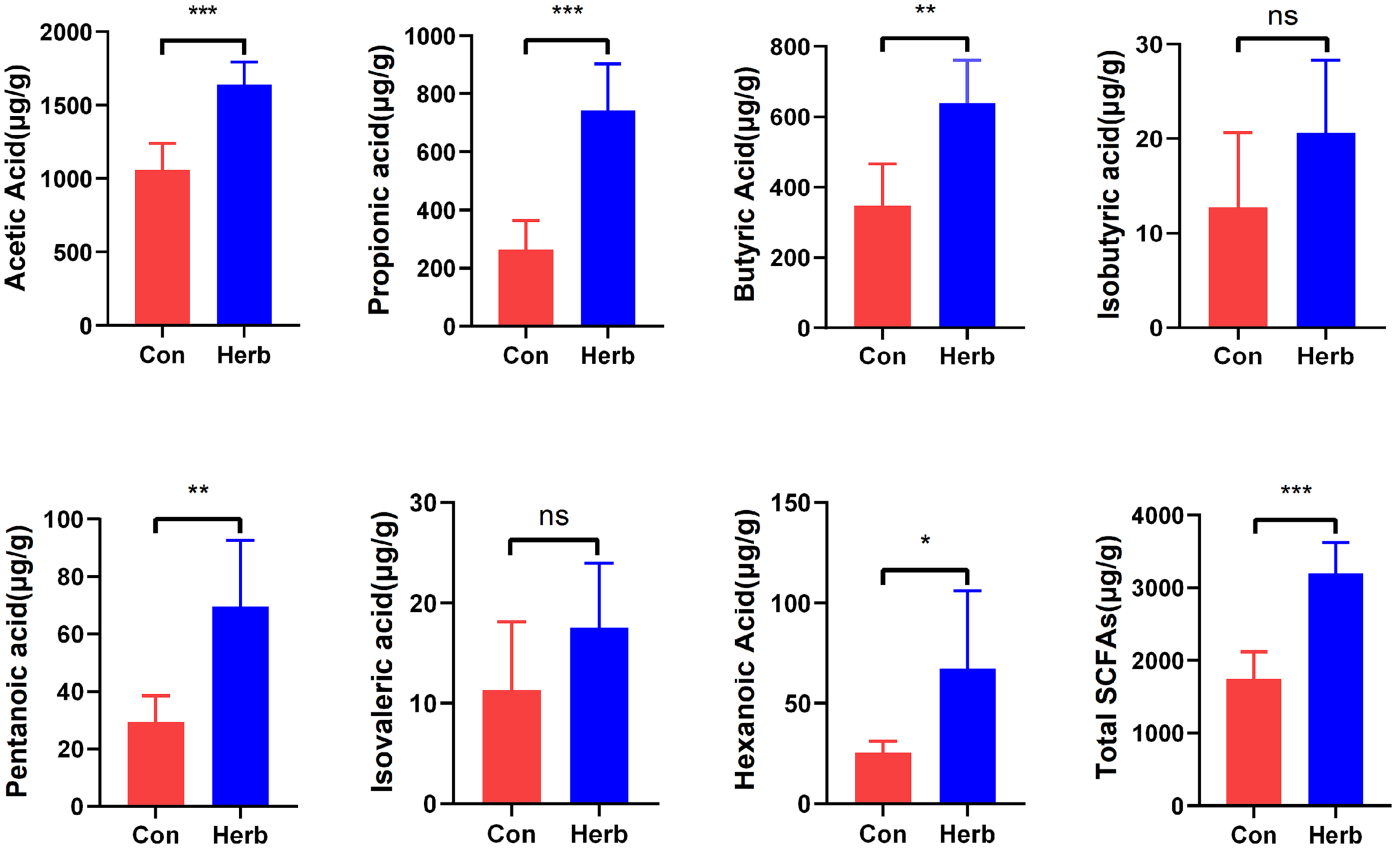
Changes in SCFAs levels in intestinal contents. The data are expressed as mean ± standard deviation: ^*^p < 0.05, ^**^p < 0.01, and ^***^p < 0.001; ns indicates *p* > 0.05.

SCFAs are important metabolites of intestinal microbiota and play a key role in regulating host metabolism, the immune system, and cell proliferation (Koh et al., 2016; Makki et al., 2018). Figure 3 depicts the effects of the long-term ingestion of purslane on SCFAs found in the colon contents of aging rats. To elaborate, acetic acid, propionic acid, and butyric acid contents were dominant. Ace, Pro, But, Pen, and Hex contents were significantly higher in the Herb group than in the Con group (*p* < 0.05). The results showed that the total SCFA content was significantly higher in the Herb group than in the Con group (*p* < 0.001).

### 3.3 Effects of purslane on the intestinal flora diversity of aging rats

**Figure 4:**
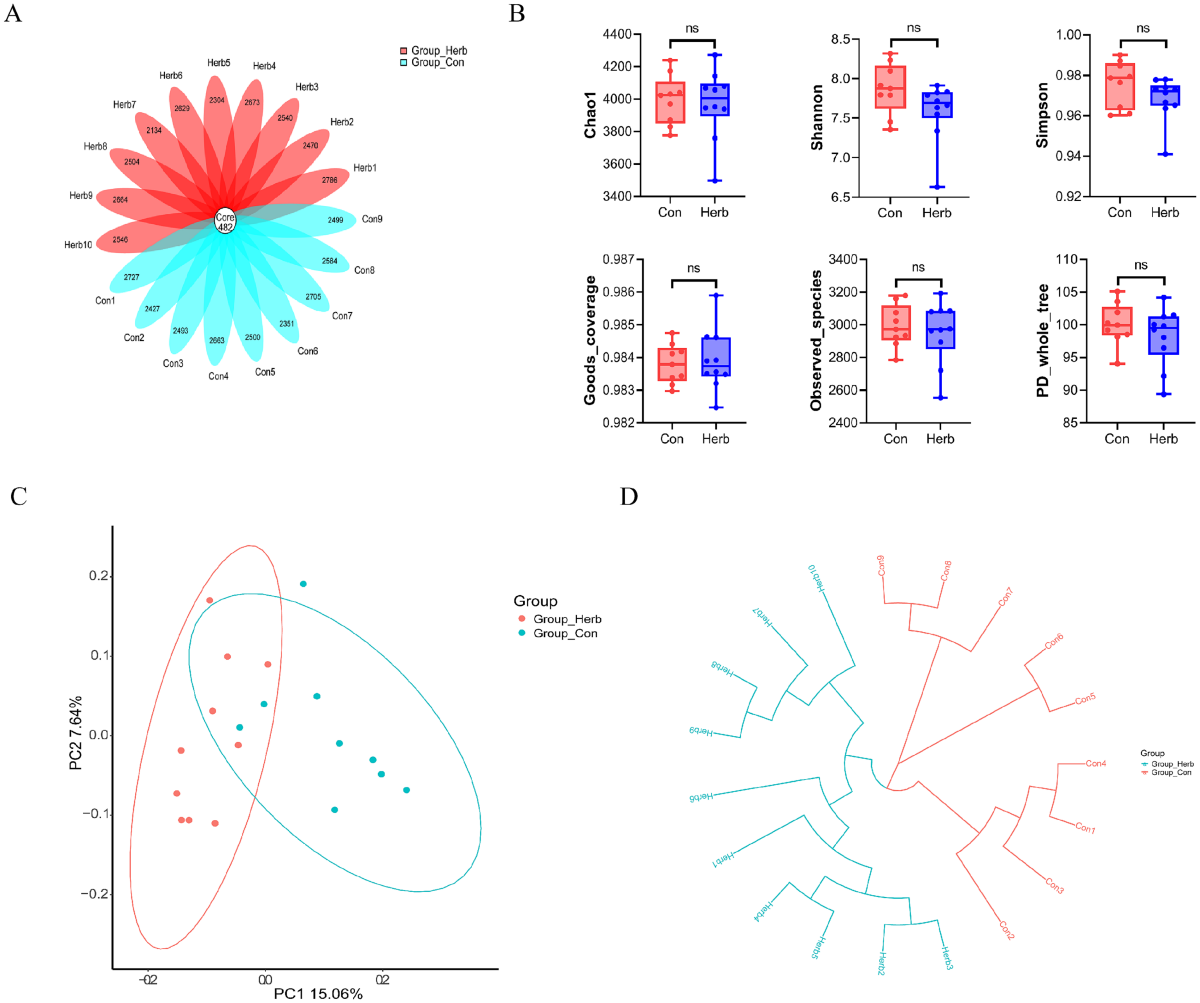
Index of bacterial diversity among the groups: **(A)** Flower plots of operational taxonomic units (OTUs) in aging rats; **(B)** Analysis of the *α* diversity of microbial flora; **(C)** *β*-diversity analysis of microbial communities using a principal coordinate analysis based on an unweighted UniFrac distance; **(D)** β-diversity analysis of microbial communities using the unweighted pair group method with arithmetic mean based on an unweighted UniFrac distance.

To investigate the molecular mechanism underlying the effects of long-term purslane supplementation on the intestinal structure and SCFA levels of aging rats, we performed 16S rRNA sequencing of fecal flora samples. Operational taxonomic units (OTUs) were annotated, and common and characteristic OTUs among the groups were indicated on a floristic map (Figure 4A). The results showed that the following *α*-diversity indexes of the two groups showed no significant differences: Chao1, Simpson, Shannon, Goods_coverage, Observed_species, and PD_whole_tree (Figure 4B). A *β*-diversity analysis of fecal communities using a principal coordinate analysis and the unweighted pair group method with arithmetic mean based on the unweighted UniFrac distance algorithm revealed that the overall microbial compositions of the Con and Herb groups were separated from each other (Figures 4C,D). These results indicate that purslane affected the intestinal flora composition of the aging rats; however, it had no effect on the abundance and diversity of the microbial communities.

### 3.4 Effects of purslane on the intestinal flora structure and composition of aging rats

**Figure 5:**
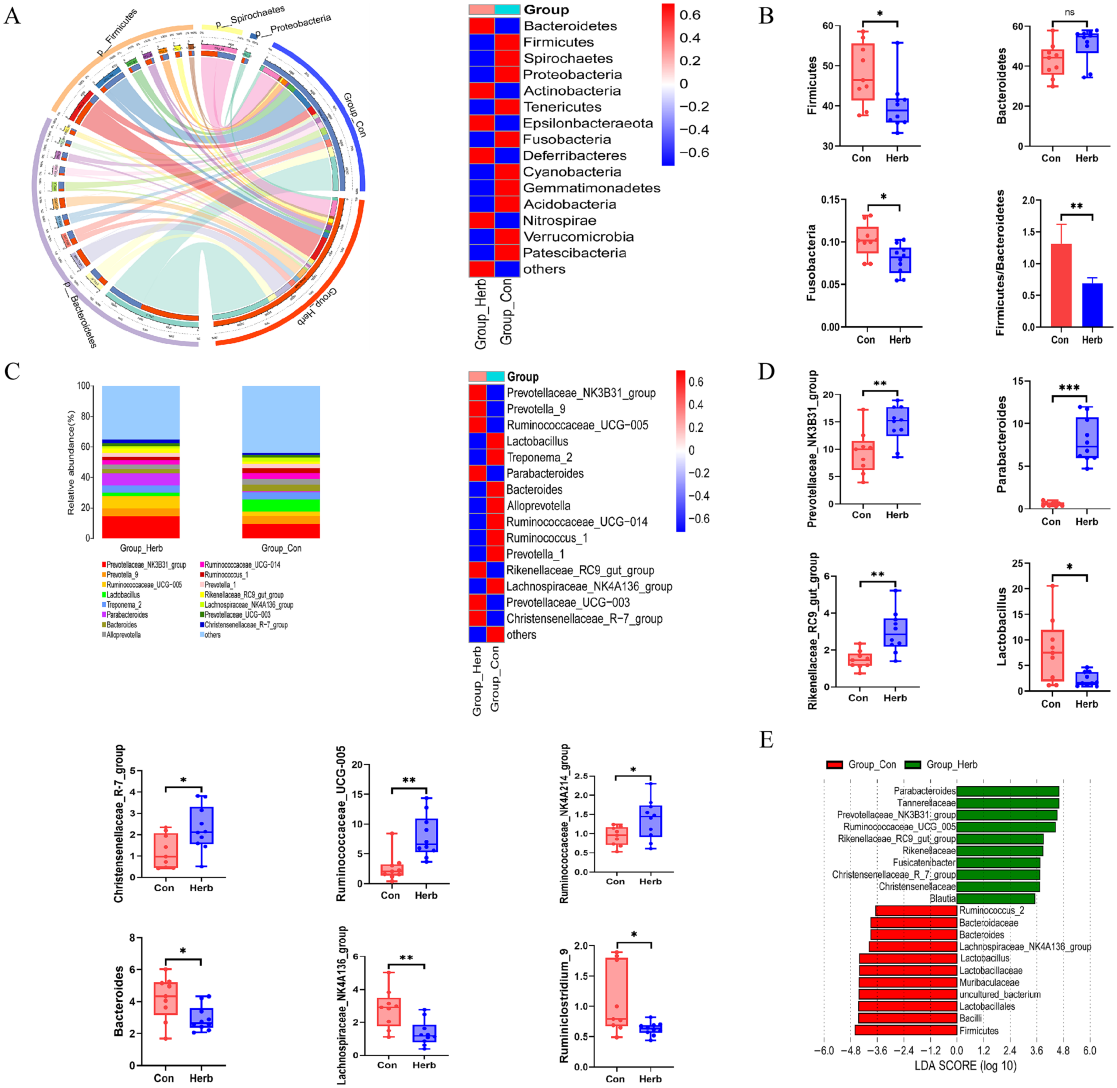
Structure and composition of intestinal flora remodeled by *Portulaca oleracea* in aging rats: **(A)** Changes in gut microbiota at the phylum level; **(B)** Changes in the relative abundance of Firmicutes, Bacteroidetes, and Fusobacteria; **(C)** Changes in gut microbiota at the genus level; **(D)** Bacteria genera with the top 10 relative abundance levels in the differential bacterial genera; **(E)** Linear discriminant analysis effect size of the different intestinal flora in the two groups—from the phylum level to the genus level.

We analyzed the OTUs of the two groups to compare the relative abundance of the bacterial communities. At the phylum level, Firmicutes and Bacteroidetes were the two most important groups in the intestinal tracts of the aging rats (Figure 5A). Compared to the Herb group, the Con group showed a significant increase in the relative abundance of Firmicutes and Fusobacteria and the ratio of Firmicutes to Bacteroidetes (F/B; *p* < 0.05) and a decrease in the relative abundance of Bacteroidetes. However, the difference was not statistically significant (Figure 5B). Figure 5C shows the proportion of the top 15 bacterial genera, with the highest relative abundance seen in both groups, whereas Figure 5D shows the top 10 relative abundance of different bacterial genera. Compared to the Con group, the Herb group exhibited a significant increase in the relative abundance of *Prevotellaceae*_NK3B31_group, *Rikenellaceae*_RC9_gut_group, *Parabacteroides*, Christensenellaceae_R-7_group, Ruminococcaceae_UCG-005, and Ruminococcaceae_NK4A214_group (*p* < 0.05) and a significant decrease in the relative abundance of Lachnospiraceae_NK4A136_group, *Lactobacillus*, *Ruminiclostridium*_9, and *Bacteroides* (*p* < 0.05). Further, the linear discriminant analysis effect size revealed that, among these genera, *Prevotellaceae*_NK3B31_group, *Rikenellaceae*_RC9_gut_group, *Parabacteroides*, *Christensenellaceae_*R-7_group, *Ruminococcaceae*_UC G-005, *Bacteroides*, *Lachnospiraceae*_NK4A136_group, and *Lactobacillus* had significant differences between the Herb and Con groups and were identified as biologically characterized groups, with a linear discriminant analysis (LDA) score of 3.5 (Figure 5E).

### 3.5 Detection of non-target metabolites in the fecal of aging rats

**Figure 6:**
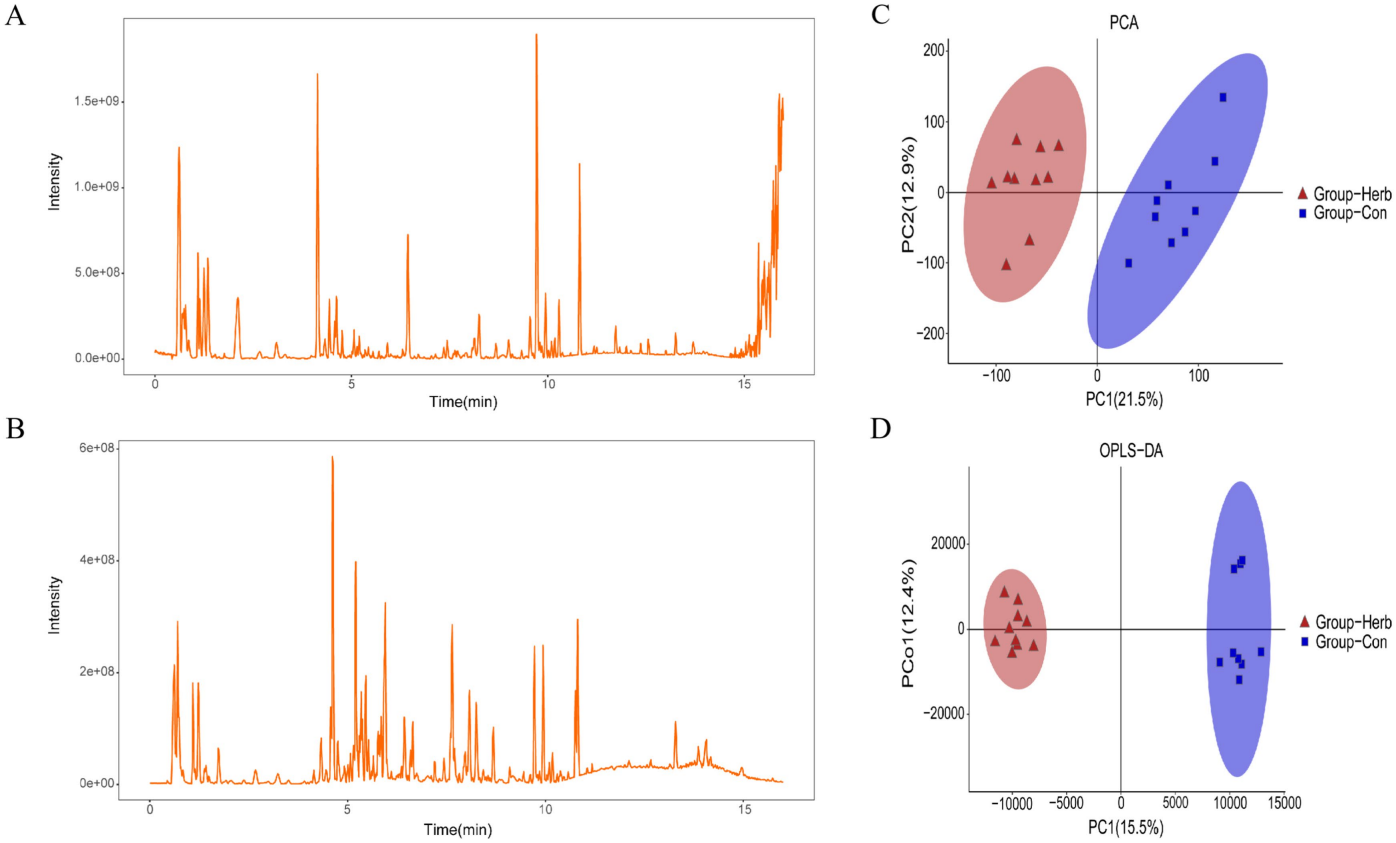
Metabolic profile analysis: **(A)** Positive ion mode current graphs; **(B)** Negative ion mode current graphs; **(C,D)** Cluster analysis of metabolites based on the principal component and orthogonal partial least squares identification method.

The total ion flow pattern of QC samples overlaps well in both the positive and negative ion modes (Figures 6A,B). In addition, the retention time, peak area, and strength of the QC samples remained stable within 48 h. The principal component and orthogonal partial least square differential analysis model were used to detect changes in metabolite clustering between groups. As can be seen in Figures 6C,D, samples from the two groups were divided into significantly different clusters, and the metabolic profiles of elderly rats underwent significant changes after long-term supplementation with purslane.

### 3.6 Analysis of differential metabolites and metabolic pathways

The important variable (VIP) in the projection is the variable weight value of the OPLS-DA model variable, which can be used to measure the influence strength and explanatory ability of different metabolite accumulation differences on the sample classification and differentiation of each group. According to the VIP value obtained from the OPLS-DA, the differences among the groups were further analyzed, and the differential metabolites were screened under these conditions: *p* < 0.05 and VIP > 1. We classified the differential metabolites in the KEGG database and identified 109 biomarkers associated with aging rats (Table 1). The metabolites in the stools of rats in the Herb group were compared with those in the Con group. The excrement of the Herb group contained L-urobilinogen. Additionally, maleic acid homopolymer, 4-[(Hydroxymethyl)nitrosoamino]-1-(3-pyridinyl)-1-butanone, and 2′-deoxyuridine were significantly upregulated (*p* < 0.01). The levels of 64 metabolites, such as 9,10,13-Trihome, 4a-Methylzymosterol-4-carboxylic acid, gamma-linolenic acid, and 9(S)-HPODE were significantly downregulated (*p* < 0.01).

**Table 1:** Differential metabolites in the Herb group as opposed to the Con group.

Metabolite	*m/z*	Formula	KEGG compound second category	Levels
*N,N′*-diacetylchitobiose	425.18	C_16_H_28_N_2_O_11_	Organooxygen compounds	↑^**^
4-O-alpha-D-Galactopyranuronosyl-D-galacturonic acid	369.07	C_12_H_18_O_13_	Organooxygen compounds	↑^**^
Isoproterenol	212.13	C_11_H_17_NO_3_	Phenols	↓_**_
N-Acetyl-L-aspartic acid	174.04	C_6_H_9_NO_5_	Carboxylic acids and derivatives	↓_***_
5-Phosphoribosylamine	274.03	C_5_H_12_NO_7_P	Organooxygen compounds	↓_**_
Lactosamine	342.14	C_12_H_23_NO_10_	Fatty acyls	↑^**^
11-Dehydro-thromboxane B2	367.21	C_20_H_32_O_6_	Fatty acyls	↓_**_
11,12-DiHETE	337.24	C_20_H_32_O_4_	Fatty acyls	↓_**_
2,3-Dinor-8-iso-PGF2alpha	309.21	C_18_H_30_O_5_	Fatty acyls	↓_**_
5-Ketoeicosatetraenoic acid	336.25	C_20_H_30_O_3_	Fatty acyls	↓_**_
5,14,15-trihydroxy-6,8,10,12-Eicosatetraenoic acid	375.21	C_20_H_32_O_5_	Fatty acyls	↓_**_
8,9-DiHETrE	383.24	C_20_H_34_O_4_	Fatty acyls	↓_**_
8,9-Epoxyeicosatrienoic acid	321.24	C_20_H_32_O_3_	Fatty acyls	↓_***_
PGB2	317.21	C_20_H_30_O_4_	Fatty acyls	↓_**_
Prostaglandin A2	379.21	C_20_H_30_O_4_	Fatty acyls	↓_**_
TXB2	371.24	C_20_H_34_O_6_	Fatty acyls	↓_**_
Prostaglandin D2	333.21	C_20_H_32_O_5_	Fatty acyls	↓_**_
12-Keto-tetrahydro-leukotriene B4	321.24	C_20_H_34_O_4_	Fatty acyls	↓_**_
Leukotriene B4	337.24	C_20_H_32_O_4_	Fatty acyls	↓_***_
Thromboxane A2	353.23	C_20_H_32_O_5_	Fatty acyls	↓_**_
(+/−)14,15-DiHETrE	339.25	C_20_H_34_O_4_	Fatty acyls	↓_**_
(2E)-N-(4-aminobutyl)-3-(4-hydroxy-3-methoxyphenyl)prop-2-enimidic acid	263.14	C_14_H_20_N_2_O_3_	Cinnamic acids and derivatives	↓_**_
4-Guanidinobutanoic acid	146.09	C_5_H_11_N_3_O_2_	Carboxylic acids and derivatives	↑^**^
Creatinine	114.07	C_4_H_7_N_3_O	Carboxylic acids and derivatives	↑^***^
N4-Acetylaminobutanal	257.15	C_6_H_11_NO_2_	Organooxygen compounds	↑^**^
Creatine	132.08	C_4_H_9_N_3_O_2_	Carboxylic acids and derivatives	↓_**_
13Z,16Z-docosadienoic acid	354.34	C_22_H_40_O_2_	Fatty Acyls	↓_**_
Alpha-Linolenic acid	296.26	C_18_H_30_O_2_	Fatty Acyls	↓_***_
2,3-Epoxyaflatoxin B1	309.04	C_17_H_12_O_7_	Coumarins and derivatives	↑^**^
4-(Methylnitrosamino)-1-(1-oxido-3-pyridinyl)-1-butanone	224.10	C_10_H_13_N_3_O_3_	Organooxygen compounds	↑^**^
4-(Methylnitrosamino)-1-(3-pyridyl)-1-butanol glucuronide	368.14	C_16_H_23_N_3_O_8_	Carboxylic acids and derivatives	↑^**^
4-(Nitrosoamino)-1-(3-pyridinyl)-1-butanone	238.08	C_9_H_11_N_3_O_2_	Organooxygen compounds	↑^**^
4-[(Hydroxymethyl)nitrosoamino]-1-(3-pyridinyl)-1-butanone	224.10	C_10_H_13_N_3_O_3_	Organooxygen compounds	↑^**^
4-Hydroxy-4-(3-pyridyl)-butanoic acid	361.14	C_9_H_11_NO_3_	Pyridines and derivatives	↓_**_
Alpha-[3-(Nitrosoamino)propyl]-3-pyridinemethanol	213.13	C_9_H_13_N_3_O_2_	Pyridines and derivatives	↑^**^
LysoPC(16:0)	540.33	C_24_H_50_NO_7_P	Glycerophospholipids	↓_**_
LysoPC(22:6(4Z,7Z,10Z,13Z,16Z,19Z))	568.34	C_30_H_50_NO_7_P	Glycerophospholipids	↓_***_
LysoPC(18:1(11Z))	522.36	C_26_H_52_NO_7_P	Glycerophospholipids	↓_**_
2-oxo-4-methylthio-butanoic acid	295.03	C_5_H_8_O_3_S	Fatty acyls	↑^**^
5′-Methylthioadenosine	280.08	C_11_H_15_N_5_O_3_S	5′-deoxyribonucleosides	↑^**^
3-Carbamoyl-2-phenylpropionic acid	227.10	C_10_H_11_NO_4_	Benzene and substituted derivatives	↓_**_
Carbamazepine iminoquinone	413.13	C_14_H_9_NO	Benzazepines	↑^**^
D-Gal alpha 1- > 6D-Gal alpha 1- > 6D-Glucose	505.18	C_18_H_32_O_16_	Organooxygen compounds	↑^**^
Melibiitol	325.11	C_12_H_24_O_11_	Fatty acyls	↑^**^
Galactosylglycerol	272.13	C_9_H_18_O_8_	Glycerolipids	↑^**^
Arbutin	290.12	C_12_H_16_O_7_	Organooxygen compounds	↑^**^
Chondroitin	424.11	C_14_H_21_NO_11_	Organooxygen compounds	↑^**^
Methylimidazole acetaldehyde	142.10	C_6_H_8_N_2_O	Azoles	↑^**^
AICAR	337.06	C_9_H_15_N_4_O_8_P	Imidazole ribonucleosides and ribonucleotides	↑^**^
(10E,12Z)-(9S)-9-Hydroperoxyoctadeca-10,12-dienoic acid	335.22	C_18_H_32_O_4_	Fatty acyls	↓_**_
9(S)-HPODE	330.26	C_18_H_32_O_4_	Fatty acyls	↓_**_
11-HpODE	623.45	C_18_H_32_O_4_	Fatty acyls	↑^**^
13-OxoODE	293.21	C_18_H_30_O_3_	Fatty acyls	↓_**_
13S-HpODE	330.26	C_18_H_32_O_4_	Fatty acyls	↑^**^
13(S)-HpODE	311.22	C_18_H_32_O_4_	Fatty acyls	↓_**_
8R-HpODE	357.23	C_18_H_32_O_4_	Fatty acyls	↓_**_
9,10,13-TriHOME	329.23	C_18_H_34_O_5_	Fatty acyls	↓_***_
Bovinic acid	279.23	C_18_H_32_O_2_	Fatty acyls	↓_**_
Arachidonic acid	305.25	C_20_H_32_O_2_	Fatty acyls	↓_***_
Gamma-Linolenic acid	279.23	C_18_H_30_O_2_	Fatty acyls	↓_***_
9(S)-HODE	295.23	C_18_H_32_O_3_	Fatty Acyls	↓_**_
Chondroitin 4-sulfate	458.06	C_14_H_23_NO_15_S	Organooxygen compounds	↓_**_
1,2-Dihydroxy-3,4-epoxy-1,2,3,4-tetrahydronaphthalene	223.06	C_10_H_10_O_3_	Tetralins	↑^**^
Morphine	303.17	C_17_H_19_NO_3_	Morphinans	↑^**^
L-Aspartic acid	134.04	C_4_H_7_NO_4_	Carboxylic acids and derivatives	↓_***_
Histamine	112.09	C_5_H_9_N_3_	Organonitrogen compounds	↑^***^
Dolichyl diphosphate	325.10	C_12_H_26_O_7_P_2_	Prenol lipids	↑^**^
Maleic acid homopolymer	287.08	C_6_H_8_O_4_	Fatty acyls	↑^**^
Phenylacetylglutamine	247.11	C_13_H_16_N_2_O_4_	Carboxylic acids and derivatives	↑^**^
3a,6b,7a,12a-Tetrahydroxy-5b-cholanoic acid	447.27	C_24_H_40_O_6_	Organooxygen compounds	↓_**_
L-Urobilinogen	597.36	C_33_H_48_N_4_O_6_	Tetrapyrroles and derivatives	↑^**^
3 alpha,7 alpha,26-Trihydroxy-5beta-cholestane	421.37	C_27_H_48_O_3_	Steroids and steroid derivatives	↓_**_
5beta-Cholestane-3alpha,7alpha,12alpha-triol	465.36	C_27_H_48_O_3_	Steroids and steroid derivatives	↓_**_
7a-Hydroxy-cholestene-3-one	401.34	C_27_H_44_O_2_	Steroids and steroid derivatives	↓_***_
Chenodeoxycholic acid	375.29	C_24_H_40_O_4_	Steroids and steroid derivatives	↑^***^
Taurochenodeoxycholic acid	517.33	C_26_H_45_NO_6_S	Steroids and steroid derivatives	↑^**^
Deoxyinosine	251.08	C_10_H_12_N_4_O_4_	Purine nucleosides	↑^**^
2’-Deoxyuridine	273.07	C_9_H_12_N_2_O_5_	Pyrimidine nucleosides	↑^**^
Cytosine	112.05	C_4_H_5_N_3_O	Diazines	↓_***_
Orotic acid	311.03	C_5_H_4_N_2_O_4_	Diazines	↑^**^
5-Amino-6-ribitylamino uracil	259.10	C_9_H_16_N_4_O_6_	Organooxygen compounds	↑^**^
5-Hydroxy-L-tryptophan	439.16	C_11_H_12_N_2_O_3_	Indoles and derivatives	↑^***^
3-ketosphinganine	300.29	C_18_H_37_NO_2_	Organooxygen compounds	↓_**_
Phytosphingosine	318.30	C_18_H_39_NO_3_	Organonitrogen compounds	↓_**_
Sphinganine 1-phosphate	420.23	C_18_H_40_NO_5_P	Sphingolipids	↓_**_
Sphingosine	300.29	C_18_H_37_NO_2_	Organonitrogen compounds	↓_**_
4a-Methylzymosterol-4-carboxylic acid	443.35	C_29_H_46_O_3_	Prenol lipids	↓_**_
4alpha-methyl-5alpha-ergosta-8,14,24(28)-trien-3beta-ol	409.35	C_29_H_44_O	Steroids and steroid derivatives	↓_**_
5-Dehydroepisterol	397.35	C_28_H_44_O	Steroids and steroid derivatives	↓_**_
7-Dehydro-desmosterol	383.33	C_27_H_42_O	Sterol Lipids	↓_**_
Obtusifoliol	443.39	C_30_H_50_O_2_	Steroids and steroid derivatives	↓_**_
Presqualene diphosphate	585.31	C_30_H_52_O_7_P_2_	Prenol lipids	↑^**^
11alpha-hydroxyprogesterone	331.23	C_21_H_30_O_3_	Sterol Lipids	↓_**_
11beta,17alpha,21-trihydroxypregnenolone	365.23	C_21_H_32_O_5_	Steroids and steroid derivatives	↓_**_
17alpha,21-Dihydroxypregnenolone	393.23	C_21_H_32_O_4_	Steroids and steroid derivatives	↓_**_
20a,22b-Dihydroxycholesterol	419.35	C_27_H_46_O_3_	Steroids and steroid derivatives	↓_**_
Alpha-Cortol	391.25	C_21_H_36_O_5_	Steroids and steroid derivatives	↓_**_
18-Hydroxycorticosterone	363.22	C_21_H_30_O_5_	Steroids and steroid derivatives	↓_**_
L-Carnitine	162.11	C_7_H_15_NO_3_	Organonitrogen compounds	↓_***_
3-Methyldioxyindole	164.07	C_9_H_9_NO_2_	Indoles and derivatives	↓_**_
Indolepyruvate	248.06	C_11_H_9_NO_3_	Indoles and derivatives	↑^**^
N-Methylserotonin	379.21	C_11_H_14_N_2_O	Indoles and derivatives	↑^**^
Indole	118.07	C_8_H_7_N	Indoles and derivatives	↓_***_
5-(L-alanin-3-yl)-2-hydroxy-cis,cis-muconate 6-semialdehyde	247.09	C_9_H_11_NO_6_	Fatty Acyls	↑^**^
Vanylglycol	185.08	C_9_H_12_O_4_	Phenols	↑^**^
3-Hydroxyphenylacetic acid	153.05	C_8_H_8_O_3_	Phenols	↑^**^
3-Polyprenyl-4-hydroxy-5-methoxybenzoate	343.13	C_18_H_24_O_4_	Benzene and substituted derivatives	↑^**^
Alpha-tocotrienol	407.33	C_29_H_44_O_2_	Prenol lipids	↓_**_
4-Pyridoxic acid	184.06	C_8_H_9_NO_4_	Pyridines and derivatives	↓_**_

“Levels” indicate the Herb group compared to the Con group; ↑ and ↓ indicate the metabolite is increased and decreased in the Herb group compared to the Con group; and **p < 0.01 and ***p < 0.001.

**Figure 7:**
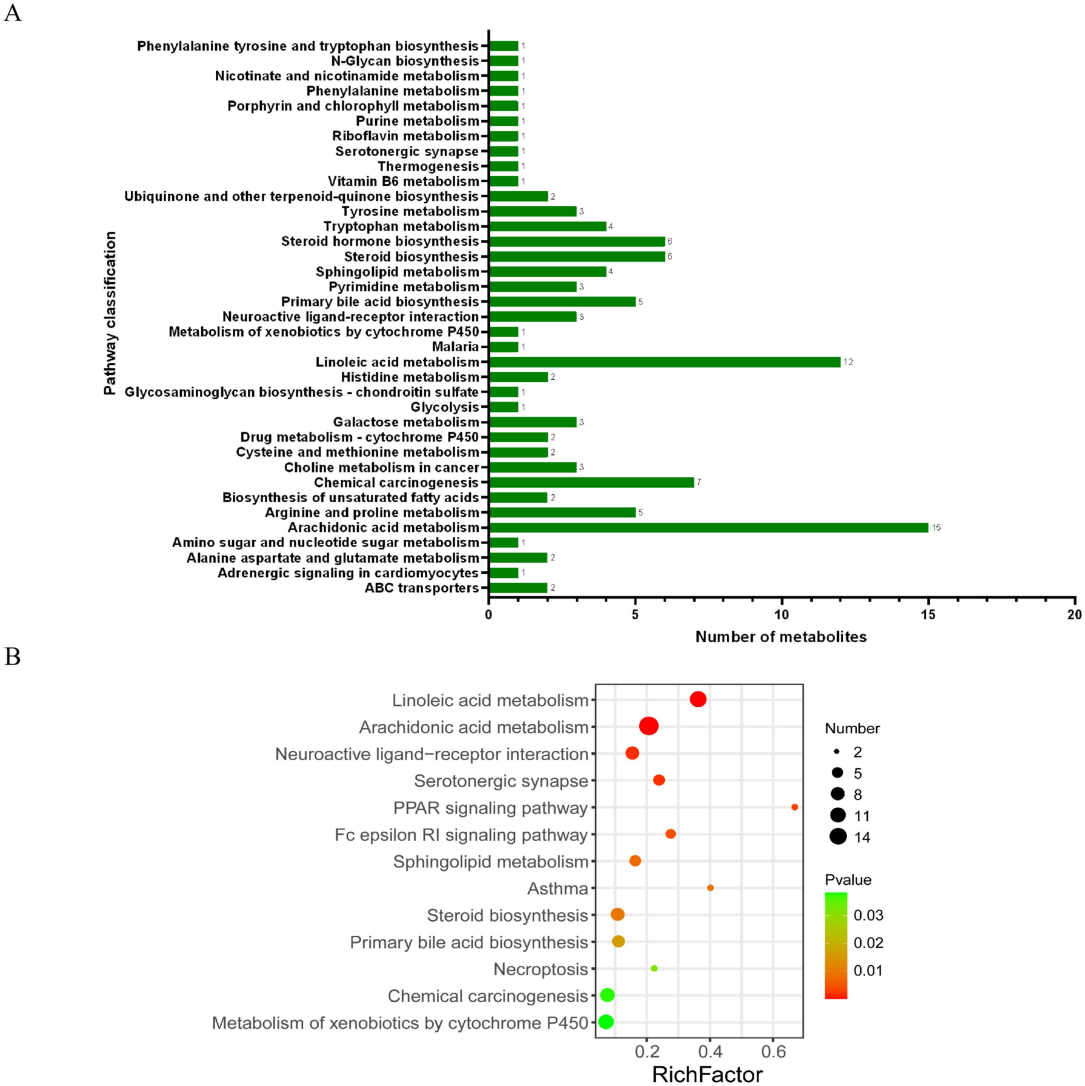
KEGG pathway analysis of differential metabolites: **(A)** KEGG functional pathway corresponds to all differential metabolites; **(B)** Bubble diagram of the KEGG topology analysis of different metabolites.

Furthermore, the KEGG analysis showed that these substances were mainly concentrated in the KEGG metabolic pathways of linoleic acid metabolism, arachidonic acid metabolism, chemical carcinogenesis, steroid biosynthesis, and steroid hormone biosynthesis (Figure 7A). In addition, the following important metabolic pathways were identified: linoleic acid metabolism, arachidonic acid metabolism, neuroactive ligand–receptor interaction, serotonergic synapse, PPAR signaling pathway, Fc epsilon RI signaling pathway, sphingolipid metabolism, asthma, steroid biosynthesis, primary bile acid biosynthesis, necroptosis, chemical carcinogenesis, and the metabolism of xenobiotics by cytochrome P450 (Figure 7B).

### 3.7 Correlations between the SCFAs and intestinal flora

**Figure 8:**
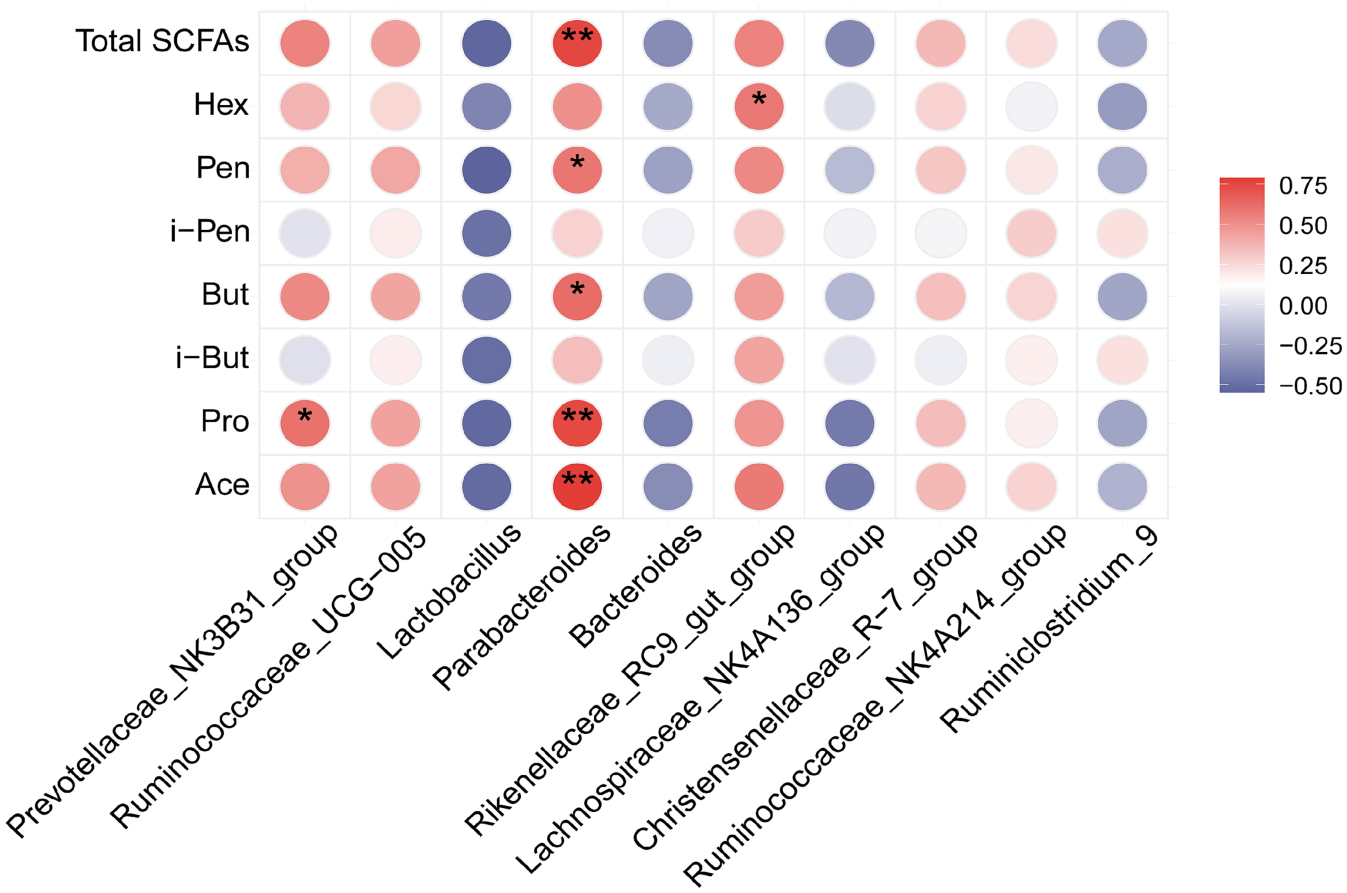
Heat map analysis of Spearman's correlation results for differentially dominant gut microbiota (top 10 genera) and SCFA parameters: Red indicates a positive correlation, while blue indicates a negative correlation; *p < 0.05 and **p < 0.01.

Spearman’s correlation analysis was conducted to determine whether there were any associations between SCFA production and changes in gut microbiota and fecal metabolites. Figure 8 shows the correlation between the top 10 relative abundance levels of different strains and each SCFA index. Notably, the SCFA indexes were significantly correlated with intestinal flora. The abundance of *Parabacteroides* was positively correlated with Ace, Pro, But, Pen, and total SCFAs. The abundance of *Prevotellaceae*_NK3B31_group and *Rikenellaceae*_RC9_gut_group was positively correlated with Pro and Hex, respectively.

## 4 Discussion

Aging can hinder the ability of intestinal epithelial tissue to continuously renew itself and can impair the protective role of epithelial cells (Teker et al., 2024). In our study, changes in the tissue morphology of the ileum and colon were observed in aging rats, and purslane alleviated these undesirable changes associated with aging. It had been reported that the consumption of nutrients could support the intestinal tract in actively conducting digestive activities, stimulating the growth and development of digestive organs (Purnasari et al., 2021). Emerging evidence suggested that purslane had a protective effect on damaged intestinal morphology and digestive activity (Yang et al., 2023).

Moreover, the main food source for intestinal epithelial cells was the SCFAs produced by the bacteria in the gut that fermented complex carbohydrates (Makki et al., 2018; Nakkarach et al., 2020). In our study, purslane increased the production of these SCFAs in the guts of older rats. In fact, Tian et al. found that purslane insoluble dietary fiber (PIDF) alleviates the toxicity of Cd mainly by increasing the production of SCFAs (Tian et al., 2021). In addition, Ning et al. showed that *Portulaca* polysaccharide could restore the contents of acetic acid and propionic acid in the intestines of mice with ulcerative colitis to the level of the normal group and that the level of butyric acid demonstrated a trend of recovery (Ning et al., 2024). These results suggest that purslane, a wild vegetable rich in complex carbohydrates, may promote the production of SCFAs in the intestines of aging bodies.

Human and animal studies had suggested that microbial changes in the aging gut in a healthy state led to increased gut inflammation and changes in host metabolism (Ling et al., 2022). As observed in our study, the richness and diversity of gut microbes in aging rats supplemented with purslane for a long time were slightly reduced; however, there was no significant difference, which is consistent with the results of Wang et al.’s studies on other animals (Wang et al., 2021). In addition, *β* diversity showed that the Con group had a large difference, and the Herb group had better cohesion, suggesting that purslane had an effect on the intestinal flora composition of aged rats. Thus, we compared the microbial communities of various groups at the taxonomic level. Our results showed that Firmicutes, Bacteroidetes, and Fusobacteria were the main dominant bacterial groups, accounting for more than 90% of the total bacteria. *Portulaca* could significantly reduce the relative abundance of Firmicutes, Fusobacteria and the ratio of F/B.

Notably, high levels of Firmicutes are associated with weight gain (Liu et al., 2024). The F/B can be used as an index to evaluate the imbalance of intestinal flora in response to various diseases (Teker et al., 2024). Recent studies had shown that purslane extract played a key role in balancing this ratio of intestinal flora and increasing the relative abundance of probiotics in mice with type 2 diabetes (Bao et al., 2022). Further, Fusobacteria are characteristic microbiota associated with a series of important chronic human diseases, including colorectal cancer (Luo et al., 2019). In the present study, purslane supplementation significantly reduced the relative abundance of Fusobacteria in aging rats. Studies involving diabetic populations had shown that *Clostridium* was negatively correlated with high dietary fiber intake (Fu et al., 2022). At the genus level, compared to the Con group, the Herb group exhibited a significant increase in the relative abundance levels of *Prevotellaceae*_NK3B31_group, *Rikenellaceae*_RC9_gut_group, *Parabacteroides*, Christensenellaceae_R-7_group, Ruminococcaceae_UCG-005, and Ruminococcaceae_NK4A214_group (*p* < 0.05) and a significant decrease in the relative abundances of Lachnospiraceae_NK4A136_group, *L.*, *Ruminiclostridium*_9, and *B.* (*p* < 0.05). *Prevotellaceae* had been shown to be related to the production of SCFAs, which regulated intestinal physiology and metabolism (Zhang et al., 2018). *Prevotellaceae*_NK3B31_group is involved in carbohydrate, amino acid, nucleotide metabolism, and lipid pathways (Zhang et al., 2018). In fact, it had been found that a high proportion of *Prevotellaceae*_NK3B31_group is beneficial for reproductive performance and intestinal health and leads to improved colostrum nutrient contents in primiparous sows (Ma et al., 2023).

The specific regulatory mechanism of *Rikenellaceae*_RC9_gut_group in the gut is still unclear. However, studies have shown that it is related to the degradation of various structural carbohydrates and the production of butyrate and other SCFAs (Weinert-Nelson et al., 2022). A significant increase in the relative abundance of Lachnospiraceae_NK4A136_group and a decrease in the relative abundance levels of *B.*, *Rikenellaceae*_RC9_gut_group, and *Parabacteroides* have been observed in bacterial diarrhea. Further, *Portulaca oleracea* extract (POE) can reconstruct the structure of the above flora (He et al., 2023). He *et al.* stated that *Parabacteroides* are characteristic flora in POE treatment and may serve as anti-inflammatory symbionts (He et al., 2023). *B.* are thought to maintain complex, beneficial relationships with their hosts; however, this relationship was not established in the present study.

Human studies had demonstrated that the transformation of microorganisms from *B.* to *Prevotellaceae*_NK3B31_group in the colon was indicative of changes in the metabolism of carbohydrates, amino acids, nucleotides, and lipids (Jiang et al., 2020). Christensenellaceae secreted *α*-arabinosidase, *β*-glucosidase, and β-galactosidase (Ma et al., 2020), and Christensenellaceae_R-7_group could regulate lipid metabolism and reduce the occurrence of obesity (Goodrich et al., 2014). Ruminococcaceae played important roles in fiber degradation and biohydrogenation (Ma et al., 2020), and Ruminococcaceae_UCG-005 could reduce dietary obesity (Zhang et al., 2019). In addition, a study showed that the recovery of the relative abundance of Ruminococcaceae_NK4A214_group was a key target for *Poria* polysaccharides in improving chronic nonbacterial prostatitis (Liu et al., 2021).

The conventional wisdom was that lactobacilli at the family and genus levels were beneficial for improving human health. However, it was worth noting that high levels of *Lactobacillaceae* had been observed in obese animals and patients (Fu et al., 2022). Studies involving Mendelian randomization analyses and animal experiments had shown that an increase in *Ruminiclostridium*_9 might be a risk factor for drug-induced and diet-related obesity (Liu et al., 2024; Li et al., 2023), as it caused abnormal lipid regulation and promotes inflammation (Zhao et al., 2021). Our data indicated that purslane might be beneficial in modulating the levels of these specific bacterial genera in the guts of aging rats.

In our study, the analysis of fecal content metabolic profiles revealed that changes in diet led to changes in metabolism. In the differential metabolite analysis, we found that purslane mainly affected the levels of 55 compounds in the feces through linoleic acid metabolism, arachidonic metabolism acid, primary bile acid biosynthesis, steroid biosynthesis, steroid hormone biosynthesis, PPAR signaling pathway, and sphingolipid metabolism pathway. The contents of taurochenodeoxycholic acid (TCDCA), chenodeoxycholic acid (CDCA), presqualene diphosphate, maleic acid homopolymer, 2-oxo-4-methylthio-butanoic acid, 11-HpODE, 13S-HpODE, lactosamine, 5-(L-alanin-3-yl)-2-hydroxy-cis, cis-muconate-6-semialdehyde, and melibiitol increased significantly, while the levels of 45 compounds including 7a-Hydroxy-cholestene-3-one decreased significantly. They belong to lipids and lipid-like molecules, organic oxygen compounds, and organic nitrogen compounds, which are involved in lipid metabolism.

A growing body of evidence suggested that changes in lipid metabolism were closely related to aging and age-related diseases (Chung, 2021). Impaired lipid accumulation and fatty acid utilization in organs were related to the pathophysiological phenotype of aging (Chung, 2021). TCDCA was one of the active substances of bile acid (BA). After dissociating into CDCA under the action of intestinal flora, it bound to the farnesol X receptor, inhibits the expression of cholesterol 7α-hydroxylase (CYP7A1), and regulated BA synthesis through negative feedback, reducing the occurrence of cholestatic diseases (Xiao et al., 2018; Rau et al., 2016). Xu *et al.* found that CDCA could promote the proliferation of porcine intestinal epithelial cells (IPEC-J2) by regulating cell cycle progression and mitochondrial function, which were beneficial to intestinal health (Xu et al., 2022). Many studies have found that using nutritional means to regulate the content of TCDCA in the body was an effective strategy to improve animal glucose and lipid metabolism, heat stress, liver injury, and growth levels (Zhang et al., 2021; Li et al., 2020; Zhang et al., 2020; Herrero-Encinas et al., 2020). Previous studies had observed that purslane could effectively balance the lipid metabolic profile in patients with metabolic syndrome (Jalali and Ghasemzadeh Rahbardar, 2022). These results suggested that purslane might promote the utilization of fatty acids in the intestines of aging rats and reduce lipid accumulation by regulating TCDCA levels to maintain intestinal health.

In addition, purslane intake resulted in increased levels of morphine, histamine, and 5-Hydroxy-L-tryptophan (5-HTP) in the feces of aging rats, while decreasing L-Aspartic acid and affecting neuroactive ligand–receptor interaction and serotonergic synapse metabolic pathways. There was consensus that the microbiome and the brain communicate with each other through multiple pathways, such as tryptophan metabolism, which included microbial metabolites (e.g., short-chain fatty acids, branched-chain amino acids, and peptidoglycans) (Wang et al., 2023). Xu et al. had found that probiotic-fermented ginseng regulates antioxidant and anti-aging activities through neuroactive ligand–receptor interaction, D-arginine, and D-ornithine metabolism in nematodes (Xu et al., 2023). In addition, studies had demonstrated the role of tryptophan and histidine in regulating energy balance (Jiao et al., 2023). Histidine could reduce body fat, appetite, oxidative stress, and systemic inflammatory markers in plasma and increase insulin sensitivity (Morris et al., 2019).

The correlation results suggested an interactive relationship between SCFAs and gut microbiota in the purslane treatment environment. In addition, the gut microbiota altered by purslane might alter host metabolism. In fact, Li et al. had demonstrated that consuming high dietary fiber increased the concentration of SCFAs and the relative abundance of *Parabacteroides* and *Rikenellaceae*_RC9_gut_group in the feces of sows during late pregnancy (Li et al., 2024). We had highlighted the positive association between *Prevotellaceae* and the production of SCFAs in the discussion section. Zhang *et al.* had found that the significant increase in the relative abundance of SCFA-producing bacteria, including *Prevotellaceae*_NK3B31_group, was consistent with the increase in SCFA levels (Zhang et al., 2024). These results suggested that *Parabacteroides*, *Prevotellaceae*_NK3B31_group, and *Rikenellaceae*_RC9_gut_group could be regulated by *Portulaca oleracea* intake to increase SCFA levels. Therefore, *Portulaca oleracea* could be used as a potential source of dietary fiber and other nutrients to improve the production of SCFAs and provide energy to intestinal cells by regulating intestinal flora and host metabolism. These results could be used as a reference for the future development of purslane products and could play a beneficial role in the promotion of healthy aging.

## 5 Conclusion

**Figure 9:**
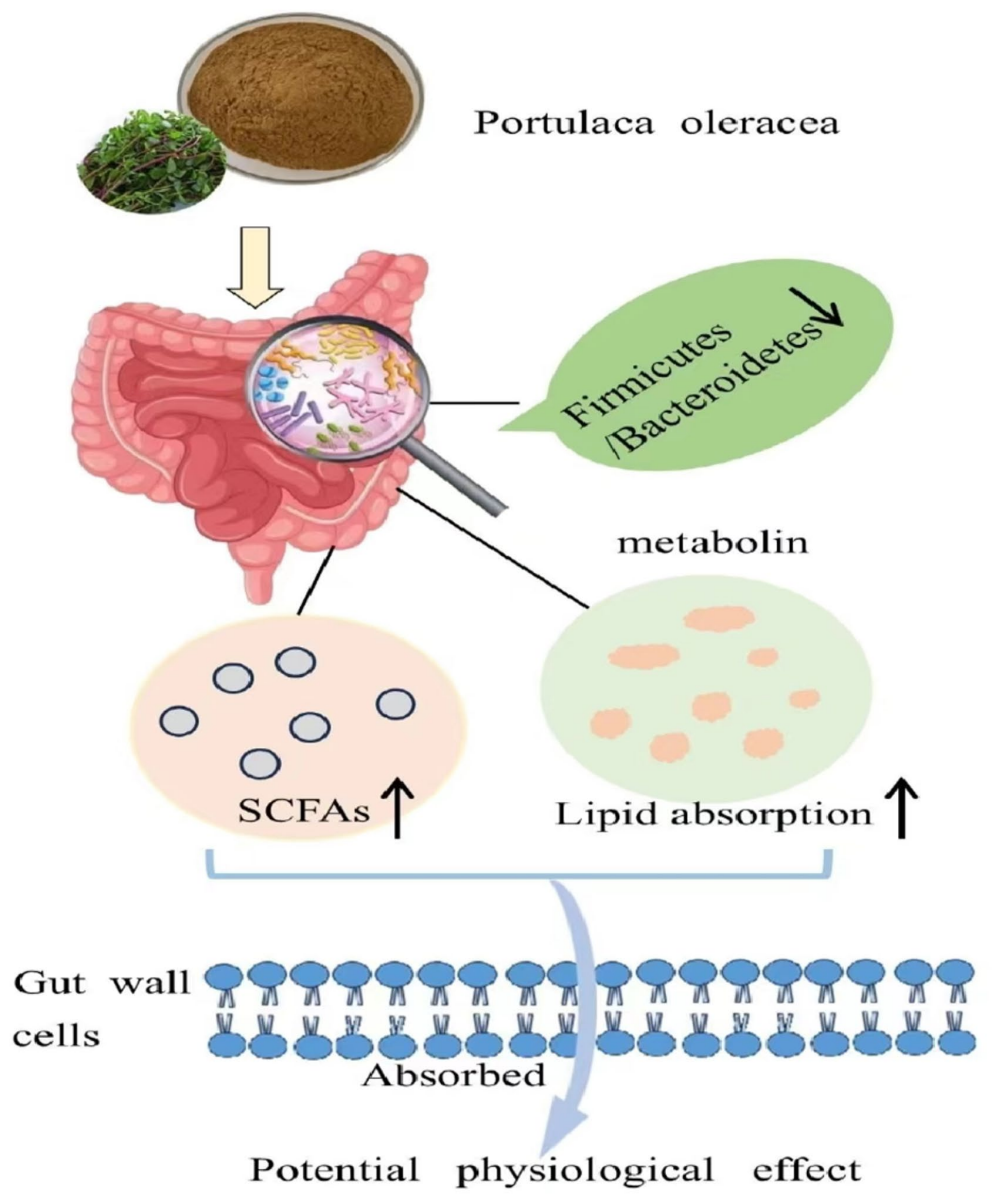
Purslane improving the aging intestinal homeostasis imbalance by reshaping the composition of intestinal flora and metabolites.

Our results vividly illustrated that purslane effectively promoted gut health in aging rats. It did so by reshaping the gut microbiota and fecal metabolites, and substantially increased the production of SCFAs, as presented in Figure 9. This not only revealed purslane’s high nutritional worth but also its potential to restore the imbalance of intestinal homeostasis in the elderly. Notably, this discovery provided a natural dietary means to improve gut health in the aging population, laid the groundwork for the development of functional foods and dietary interventions targeting age-related gut problems.

## Data Availability

The datasets presented in this study can be found in online repositories. The names of the repository/repositories and accession number(s) can be found in the article/supplementary material.

## Footnotes

^1^ https://www.genome.jp/kegg/pathway.html

^2^ https://hmdb.ca

^3^ https://www.lipidmaps.org

## References

[ref1] Bao M. Hou K. Xin C. Zeng D. Cheng C. Zhao H.. (2022). Extract alleviated type 2 diabetes via modulating the gut microbiota and serum branched-chain amino acid metabolism. Mol. Nutr. Food Res. 66:e2101030. doi: 10.1002/mnfr.202101030, PMID: 35212446

[ref2] Bruins M. J. Van Dael P. Eggersdorfer M. (2019). The role of nutrients in reducing the risk for noncommunicable diseases during aging. Nutrients 11:85. doi: 10.3390/nu11010085, PMID: 30621135 PMC6356205

[ref3] Chung K. W. (2021). Advances in understanding of the role of lipid metabolism in aging. Cells 10:880. doi: 10.3390/cells10040880, PMID: 33924316 PMC8068994

[ref4] Coman V. Vodnar D. C. (2020). Gut microbiota and old age: modulating factors and interventions for healthy longevity. Exp. Gerontol. 141:111095. doi: 10.1016/j.exger.2020.111095, PMID: 32979504 PMC7510636

[ref5] da Costa J. P. Vitorino R. Silva G. M. Vogel C. Duarte A. C. Rocha-Santos T. (2016). A synopsis on aging-theories, mechanisms and future prospects. Ageing Res. Rev. 29, 90–112. doi: 10.1016/j.arr.2016.06.005, PMID: 27353257 PMC5991498

[ref6] Fang E. F. Scheibye-Knudsen M. Jahn H. J. Li J. Ling L. Guo H.. (2015). A research agenda for aging in China in the 21st century. Ageing Res. Rev. 24, 197–205. doi: 10.1016/j.arr.2015.08.003, PMID: 26304837 PMC5179143

[ref7] Fu Q. Huang H. Ding A. W. Yu Z. Q. Huang Y. P. Fu G. P.. (2022). Polysaccharides reduce serum lipid levels in aging rats by modulating intestinal microbiota and metabolites. Front. Nutr. 9:9. doi: 10.3389/fnut.2022.965653, PMID: 35983485 PMC9378863

[ref8] Fu J. Xu K. Ni X. Li X. Zhu X. Xu W. (2022). Habitual dietary Fiber intake, fecal microbiota, and hemoglobin A1c level in Chinese patients with type 2 diabetes. Nutrients 14:1003. doi: 10.3390/nu14051003, PMID: 35267978 PMC8912884

[ref9] Goodrich J. K. Waters J. L. Poole A. C. Sutter J. L. Koren O. Blekhman R.. (2014). Human genetics shape the gut microbiome. Cell 159, 789–799. doi: 10.1016/j.cell.2014.09.053, PMID: 25417156 PMC4255478

[ref10] He Y. Long H. Zou C. Yang W. Jiang L. Xiao Z.. (2021). Anti-nociceptive effect of *Portulaca oleracea L*. ethanol extracts attenuated zymosan-induced mouse joint inflammation via inhibition of Nrf2 expression. Innate Immun. 27, 230–239. doi: 10.1177/1753425921994190, PMID: 33611955 PMC8054150

[ref11] He Y. Xu G. Jiang P. She D. Huang L. Chen C. (2023). Antibacterial diarrhea effect and action mechanism of *Portulaca oleracea L.* water extract based on the regulation of gut microbiota and fecal metabolism. J. Sci. Food Agric. 103, 7260–7272. doi: 10.1002/jsfa.12810, PMID: 37357594

[ref12] Herrero-Encinas J. Blanch M. Pastor J. J. Mereu A. Ipharraguerre I. R. Menoyo D. (2020). Effects of a bioactive olive pomace extract from *Olea europaea* on growth performance, gut function, and intestinal microbiota in broiler chickens. Poult. Sci. 99, 2–10. doi: 10.3382/ps/pez467, PMID: 32416802 PMC7587805

[ref13] Jalali J. Ghasemzadeh Rahbardar M. (2022). Ameliorative effects of *Portulaca oleracea L.* (purslane) on the metabolic syndrome: a review. J. Ethnopharmacol. 299:115672. doi: 10.1016/j.jep.2022.115672, PMID: 36064150

[ref14] Jiang X. Lu N. Zhao H. Yuan H. Xia D. Lei H. (2020). The microbiome-metabolome response in the Colon of piglets under the status of weaning stress. Front. Microbiol. 11:2055. doi: 10.3389/fmicb.2020.02055, PMID: 32983040 PMC7483555

[ref15] Jiao W. Sang Y. Wang X. Wang S. (2023). Metabonomics and the gut microbiome analysis of the effect of 6-shogaol on improving obesity. Food Chem. 404:134734. 36327507 10.1016/j.foodchem.2022.134734

[ref16] Koh A. De Vadder F. Kovatcheva-Datchary P. Backhed F. (2016). From dietary Fiber to host physiology: short-chain fatty acids as key bacterial metabolites. Cell 165, 1332–1345. doi: 10.1016/j.cell.2016.05.041, PMID: 27259147

[ref17] Lee J. d'Aigle J. Atadja L. Quaicoe V. Honarpisheh P. Ganesh B. P.. (2020). Gut microbiota-derived short-chain fatty acids promote Poststroke recovery in aged mice. Circ. Res. 127, 453–465. doi: 10.1161/CIRCRESAHA.119.316448, PMID: 32354259 PMC7415518

[ref18] Li Y. He J. Zhang L. Liu H. Cao M. Lin Y.. (2024). Improvement of insulin sensitivity by dietary fiber consumption during late pregnant sows is associated with gut microbiota regulation of tryptophan metabolism. Animal Microbiome 6:34. 38907293 10.1186/s42523-024-00323-6 PMC11191243

[ref19] Li S. Li S. Liu S. Lu S. Li J. Cheng S.. (2024). *Portulaca oleracea* exhibited anti-coccidian activity, fortified the gut microbiota of Hu lambs. AMB Express 14:50. doi: 10.1186/s13568-024-01705-4, PMID: 38700828 PMC11068709

[ref20] Li X. Xiao Y. Song L. Huang Y. Chu Q. Zhu S.. (2020). Effect of *Lactobacillus plantarum* HT121 on serum lipid profile, gut microbiota, and liver transcriptome and metabolomics in a high-cholesterol diet-induced hypercholesterolemia rat model. Nutrition 79-80:110966. doi: 10.1016/j.nut.2020.110966 32942130

[ref21] Li S. N. Zhang D. L. Wang Z. H. Song W. T. Chen W. B. Hu G. L.. (2023). Anti-obesity effects exerted by *Dioscorea opposita* Thunb. Polysaccharides in diet-induced obese mice. Food Sci. Nutr. 11, 6459–6469. doi: 10.1002/fsn3.3588, PMID: 37823169 PMC10563686

[ref22] Ling Z. Liu X. Cheng Y. Yan X. Wu S. (2022). Gut microbiota and aging. Crit. Rev. Food Sci. Nutr. 62, 3509–3534. doi: 10.1080/10408398.2020.1867054, PMID: 33377391

[ref23] Liu S. Li F. Cai Y. Ren L. Sun L. Gang X.. (2024). Unraveling the mystery: a Mendelian randomized exploration of gut microbiota and different types of obesity. Front. Cell. Infect. Microbiol. 14:1352109. doi: 10.3389/fcimb.2024.1352109, PMID: 38375360 PMC10875079

[ref24] Liu J. Liu L. Zhang G. Peng X. (2021). Poria cocos polysaccharides attenuate chronic nonbacterial prostatitis by targeting the gut microbiota: comparative study of Poria cocos polysaccharides and finasteride in treating chronic prostatitis. Int. J. Biol. Macromol. 189, 346–355. doi: 10.1016/j.ijbiomac.2021.08.139, PMID: 34428489

[ref25] Luo K. Zhang Y. Xv C. Ji J. Lou G. Guo X.. (2019). *Fusobacterium nucleatum*, the communication with colorectal cancer. Biomed. Pharmacother 116:108988. doi: 10.1016/j.biopha.2019.108988, PMID: 31112873

[ref26] Lv W. J. Huang J. Y. Li S. P. Gong X. P. Sun J. B. Mao W.. (2022). Extracts alleviate 2,4-dinitrochlorobenzene-induced atopic dermatitis in mice. Front. Nutr. 9:986943. doi: 10.3389/fnut.2022.986943 36051905 PMC9424637

[ref27] Ma Z. Wu Z. Wang Y. Meng Q. Chen P. Li J.. (2023). Effect of yeast culture on reproductive performance, gut microbiota, and Milk composition in Primiparous sows. Animals 13:2954. doi: 10.3390/ani13182954, PMID: 37760354 PMC10525930

[ref28] Ma J. Zhu Y. Wang Z. Yu X. Hu R. Wang X.. (2020). Comparing the bacterial Community in the Gastrointestinal Tracts between Growth-Retarded and Normal Yaks on the Qinghai-Tibetan plateau. Front. Microbiol. 11:600516. doi: 10.3389/fmicb.2020.600516, PMID: 33391217 PMC7775487

[ref29] Makki K. Deehan E. C. Walter J. Backhed F. (2018). The impact of dietary Fiber on gut microbiota in host health and disease. Cell Host Microbe 23, 705–715. doi: 10.1016/j.chom.2018.05.012 29902436

[ref30] Morris J. K. Piccolo B. D. John C. S. Green Z. D. Thyfault J. P. Adams S. H. (2019). Oxylipin profiling of Alzheimer's disease in nondiabetic and type 2 diabetic elderly. Meta 9:177. doi: 10.3390/metabo9090177, PMID: 31491971 PMC6780570

[ref31] Morrison D. J. Preston T. (2016). Formation of short chain fatty acids by the gut microbiota and their impact on human metabolism. Gut Microbes 7, 189–200. doi: 10.1080/19490976.2015.1134082, PMID: 26963409 PMC4939913

[ref32] Mou Y. Du Y. Zhou L. X. Yue J. R. Hu X. L. Liu Y. X.. (2022). Gut microbiota interact with the brain through systemic chronic inflammation: implications on Neuroinflammation, neurodegeneration, and aging. Front. Immunol. 13:13. doi: 10.3389/fimmu.2022.796288 PMC9021448 35464431

[ref33] Nakkarach A. Foo H. L. Song A. A. Nitisinprasert S. Withayagiat U. (2020). Promising discovery of beneficial *Escherichia coli* in the human gut. 3 Biotech 10:296. doi: 10.1007/s13205-020-02289-z, PMID: 32550113 PMC7283410

[ref34] Ning K. Shi C. Chi Y. Y. Zhou Y. F. Zheng W. Duan Y.. (2024). *Portulaca oleracea* L. Polysaccharide alleviates dextran sulfate sodium-induced ulcerative colitis by regulating intestinal homeostasis. Int. J. Biol. Macromol. 256:128375. doi: 10.1016/j.ijbiomac.2023.128375 38000581

[ref35] Purnasari P. W. Nasihun T. Zulaikhah S. T. (2021). Effects of single or combined supplementation of probiotics and zinc on histological features of ileum, glucagon like Peptide-1 and ghrelin levels in malnourished rats. Folia Med. 63, 59–66. doi: 10.3897/folmed.63.e53768, PMID: 33650397

[ref36] Pyo I. S. Yun S. Yoon Y. E. Choi J. W. Lee S. J. (2020). Mechanisms of aging and the preventive effects of resveratrol on age-related diseases. Molecules 25:20. doi: 10.3390/molecules25204649, PMID: 33053864 PMC7587336

[ref37] Rau M. Stieger B. Monte M. J. Schmitt J. Jahn D. Frey-Wagner I.. (2016). Alterations in enterohepatic Fgf15 signaling and changes in bile acid composition depend on localization of murine intestinal inflammation. Inflamm. Bowel Dis. 22, 2382–2389. doi: 10.1097/MIB.0000000000000879, PMID: 27580383

[ref38] Schoeler M. Caesar R. (2019). Dietary lipids, gut microbiota and lipid metabolism. Rev. Endocr. Metab. Disord. 20, 461–472. doi: 10.1007/s11154-019-09512-0, PMID: 31707624 PMC6938793

[ref39] Shilpa Dogra D. D. Sugiyama T. Stathi A. Gardiner P. A. Owen N. (2022). Active aging and public health: evidence, implications, and opportunities. Annu. Rev. Public Health 43, 439–459. doi: 10.1146/annurev-publhealth-052620-091107 34910580

[ref40] Sun M. F. Shen Y. Q. (2018). Dysbiosis of gut microbiota and microbial metabolites in Parkinson's disease. Ageing Res. Rev. 45, 53–61. doi: 10.1016/j.arr.2018.04.004, PMID: 29705121

[ref41] Teker H. T. Ceylani T. Keskin S. Samgane G. Allahverdi H. Acikgoz E.. (2024). Supplementing probiotics during intermittent fasting proves more effective in restoring ileum and colon tissues in aged rats. J. Cell. Mol. Med. 28:e18203. doi: 10.1111/jcmm.18203, PMID: 38445809 PMC10915827

[ref42] Tian X. Ding Y. Kong Y. Wang G. Wang S. Cheng D. (2021). Purslane (*Portulacae oleracea L*.) attenuates cadmium-induced hepatorenal and colonic damage in mice: role of chelation, antioxidant and intestinal microecological regulation. Phytomedicine 92:153716. doi: 10.1016/j.phymed.2021.153716, PMID: 34481339

[ref43] Wang C. Liu Q. Ye F. Tang H. Xiong Y. Wu Y.. (2021). Dietary purslane (*Portulaca oleracea L*.) promotes the growth performance of broilers by modulation of gut microbiota. AMB Express 11:31. doi: 10.1186/s13568-021-01190-z, PMID: 33620605 PMC7902751

[ref44] Wang Q. Yang Q. Liu X. (2023). The microbiota-gut-brain axis and neurodevelopmental disorders. Protein Cell 14, 762–775. doi: 10.1093/procel/pwad026 37166201 PMC10599644

[ref45] Weinert-Nelson J. R. Biddle A. S. Williams C. A. (2022). Fecal microbiome of horses transitioning between warm-season and cool-season grass pasture within integrated rotational grazing systems. Anim. Microbiome 4:41. doi: 10.1186/s42523-022-00192-x, PMID: 35729677 PMC9210719

[ref46] Xiao Y. Zhou K. Lu Y. Yan W. Cai W. Wang Y. (2018). Administration of antibiotics contributes to cholestasis in pediatric patients with intestinal failure via the alteration of FXR signaling. Exp. Mol. Med. 50, 1–14. doi: 10.1038/s12276-018-0181-3, PMID: 30504803 PMC6269533

[ref47] Xu L. Li Y. Wei Z. Bai R. Gao G. Sun W.. (2022). Chenodeoxycholic acid (CDCA) promoted intestinal epithelial cell proliferation by regulating cell cycle progression and mitochondrial biogenesis in IPEC-J2 cells. Antioxidants 11:2285. doi: 10.3390/antiox11112285, PMID: 36421471 PMC9687205

[ref48] Xu H. Y. Li Q. C. Zhou W. J. Zhang H. B. Chen Z. X. Peng N.. (2023). Anti-oxidative and anti-aging effects of probiotic fermented ginseng by modulating gut microbiota and metabolites in *Caenorhabditis elegans*. Plant Foods Hum. Nutr. 78, 320–328. doi: 10.1007/s11130-023-01055-9, PMID: 36947370

[ref49] Yang S. Feng L. Zhang J. Yan C. Zhang C. Huang Y.. (2023). Digestion activity and microbiome of Chinese pond turtle (*Mauremys reevesii*) during *Aeromonas hydrophila* infection. Int. J. Mol. Sci. 24:12. doi: 10.3390/ijms241210260, PMID: 37373406 PMC10298896

[ref50] Zhang C. Gan Y. Lv J. W. Qin M. Q. Hu W. R. Liu Z. B.. (2020). The protective effect of obeticholic acid on lipopolysaccharide-induced disorder of maternal bile acid metabolism in pregnant mice. Int. Immunopharmacol. 83:106442. doi: 10.1016/j.intimp.2020.106442, PMID: 32248018

[ref51] Zhang S. Y. Li R. J. W. Lim Y. M. Batchuluun B. Liu H. Waise T. M. Z.. (2021). FXR in the dorsal vagal complex is sufficient and necessary for upper small intestinal microbiome-mediated changes of TCDCA to alter insulin action in rats. Gut 70, 1675–1683. doi: 10.1136/gutjnl-2020-321757, PMID: 33087489

[ref52] Zhang L. Ouyang Y. Li H. Shen L. Ni Y. Fang Q.. (2019). Metabolic phenotypes and the gut microbiota in response to dietary resistant starch type 2 in normal-weight subjects: a randomized crossover trial. Sci. Rep. 9:4736. doi: 10.1038/s41598-018-38216-9, PMID: 30894560 PMC6426958

[ref53] Zhang Y. Wang C. Lang H. Yu H. Zhou M. Rao X.. (2024). The contrasting effects of two distinct exercise training modalities on exhaustive exercise-induced muscle damage in mice may be associated with alterations in the gut microbiota. Int. J. Mol. Sci. 25:7837. doi: 10.3390/ijms25147837 39063080 PMC11277320

[ref54] Zhang L. Wu W. Lee Y. K. Xie J. Zhang H. (2018). Spatial heterogeneity and co-occurrence of mucosal and luminal microbiome across swine intestinal tract. Front. Microbiol. 9:48. doi: 10.3389/fmicb.2018.00048, PMID: 29472900 PMC5810300

[ref55] Zhao Q. Hou D. Fu Y. Xue Y. Guan X. Shen Q. (2021). Adzuki bean alleviates obesity and insulin resistance induced by a high-fat diet and modulates gut microbiota in mice. Nutrients 13:3240. doi: 10.3390/nu13093240, PMID: 34579118 PMC8466346

[ref56] Zhou Y. X. Xin H. L. Rahman K. Wang S. J. Peng C. Zhang H. (2015). *Portulaca oleracea L*.: a review of phytochemistry and pharmacological effects. Biomed. Res. Int. 2015:925631, 1–11. doi: 10.1155/2015/925631 25692148 PMC4321094

